# Multi-omics Identify the Role of FGF7 Pathway in Diabetic Foot Ulcers

**DOI:** 10.5812/ijpr-162294

**Published:** 2025-09-15

**Authors:** Wenkang Luan, Shujun Fan, Dongwen Jiang, Leren He

**Affiliations:** 1 Department of Auricular Reconstruction, Plastic Surgery Hospital, Chinese Academy of Medical Sciences, Peking Union Medical College, Beijing, China

**Keywords:** FGF7, Diabetic Foot Ulcers, Weighted Co-expression Network Analysis, Single-Cell Analysis, Intercellular Communication Analysis

## Abstract

**Background:**

Diabetic foot ulcers (DFUs) are one of the most common and serious complications of diabetes.

**Objectives:**

The objective of the study is to identify key genes and cellular mechanisms driving DFU pathogenesis and healing using multi-omics integration.

**Methods:**

We used differential expression analysis and weighted co-expression network analysis (WGCNA) to identify key genes in DFU. We constructed protein-protein interaction (PPI) networks through STRING and Cytoscape. Support vector machine-recursive feature elimination (SVM-RFE) was used to screen out potential diagnostic biomarkers. Single-cell transcriptomic analysis detected differences in the cellular landscape, and intercellular communication analysis deciphered the key intercellular signaling pathway.

**Results:**

We first found 388 differentially expressed genes that are closely related to DFU (fold change > 2 and WGCNA-derived module significantly correlated with DFU, R = 0.78). We further constructed a PPI network and identified 15 hub genes and 10 diagnostic biomarkers (including FGF7) for DFU. FGF7 is lowly expressed in DFU and enriched in stromal cells and fibroblasts in DFU, and participates in the immune microenvironment of DFU. FGF7-FGFR1 is the main pathway for intercellular communication involving fibroblasts and stromal cells in the healing process of DFU.

**Conclusions:**

These results provide an in-depth understanding of the multifactorial mechanisms underlying DFU progression and healing, offering a theoretical basis for optimizing clinical treatment.

## 1. Background

Diabetic foot ulcers (DFUs) are a type of foot infection, ulceration, and even deep tissue destruction in the distal lower extremities of diabetic patients (1). The global prevalence of DFU is about 6.3%, and it is expected that close to 25% of diabetic patients will develop DFU in their lifetimes (2-5). The DFU seriously affect the quality of life, leading to prolonged hospitalization and even lower extremity amputation or death (6, 7). The 5-year mortality rate for patients undergoing amputation for DFU is more than 50%, which is much higher than most tumors (8). Although the healing rate for DFU after 12 weeks of treatment is 24 - 82%, the prognosis for DFU is poor, with recurrence rates as high as 60% (9, 10).

The wound-healing process in patients with diabetes is impaired by multiple factors, leading to the development of chronic wounds (9, 11). Many cell types — including fibroblasts, keratinocytes, stromal cells, and immune cells — play critical roles in distinct healing stages (12-15). Intercellular communication enables multiple cells to coordinate with each other and complete various biological tasks, such as wound healing, where many different types of cells participate through this communication and bioactive factors (16-20). However, dysregulation of cellular crosstalk in DFU remains incompletely understood.

The DFU are now recognized as a complex disease determined by a combination of genetic and environmental factors. Although some studies have reported that the imbalance of some growth factors and inflammatory factors and the change of the extracellular matrix are closely related to DFU (21-24), the role of genetics in DFU remains uncertain. In recent years, research on transcriptome and single-cell sequencing and related bioinformatics analysis has played an important role in clarifying the pathogenesis and healing mechanism of DFU. Specifically, some scholars used transcriptome data to identify differentially expressed genes in DFU (25). Others have identified angiogenesis-related genes and immune-related genes in DFU (26, 27). However, these studies lack multi-dimensional analysis to explore the driving genes for the formation and healing of DFU.

## 2. Objectives

In this paper, we combined transcriptome sequencing analysis, weighted co-expression network analysis (WGCNA) analysis, single-cell sequencing analysis, machine learning algorithms, immune cell analysis, and histological verification of tissue samples to identify the key genes in DFU. We found that FGF7 is the sole hub gene (a gene that occupies a central position in a biological network) identified as a diagnostic biomarker (a biological characteristic that objectively indicates normal/pathological processes) for DFU. FGF7, a member of the fibroblast growth factor (FGF) family, has various biological functions such as regulating cell differentiation and inhibiting cell apoptosis (28, 29). Some scholars have explored the application of the FGF family in therapy to promote the healing of chronic wounds (30). Here, we further reveal the role of FGF7 in DFU through multi-dimensional transcriptomic analysis. Moreover, we analyzed the single-cell transcriptomic datasets and found that DFU healers had a higher proportion of stromal cells compared to DFU non-healers. Additionally, FGF7 is mainly expressed in stromal cells and fibroblasts in DFU healers and enriched in fibroblasts in DFU non-healers. We further found that FGF7-FGFR1 is the potential main pathway for intercellular communication involving fibroblasts and stromal cells in the healing process of DFU. These results provide a more comprehensive understanding of DFU and its healing process.

## 3. Methods

### 3.1. Data Acquisition

We visited The Gene Expression Omnibus (GEO) website to search for the term "diabetic foot ulcer". We found that only the GEO#GSE80178 dataset met the criteria, which included 3 diabetic foot skin (DFS) tissues, 6 DFU tissues, and 3 non-diabetic foot skin (NDFS) tissues. Next, we searched for the keywords "diabetic foot ulcer" and "single cell". We found only a single-cell transcriptomic GEO#GSE165816 dataset, which included 10 non-diabetic subjects and 17 diabetic patients (11 patients with DFU and 6 without DFU).

### 3.2. Differential Expression Analysis

We analyzed genomic profiling of 3 DFS tissues and 6 DFU tissues in the GSE80178 DFU datasets to identify differentially expressed genes (DEG) of DFU. We used the NormalizeBetweenArrays algorithm to correct and normalize the data. When the changes in gene expression between the two groups were more than twofold and the adjusted P-value was less than 0.05, this gene was considered to be a DEG. We used the pheatmap and ggplot2 packages in the R project to draw a heat map and volcano plot of DEG in DFU.

### 3.3. Weighted Co-expression Network Analysis Analysis

The R package termed “WGCNA” was used to conduct WGCNA analysis of the GSE80178 datasets. For correcting and normalizing the data, the NormalizeBetweenArrays algorithm was used. If there are genes that appear multiple times, the average expression is taken. A total of 9376 genes were included for analysis. We clustered the samples, removed outliers, and then set appropriate soft thresholds to construct a scale-free network. Next, the adjacency matrix and topological overlap matrix were constructed, and hierarchical clustering was used to identify modules (including at least 60 genes). Then, we calculated the eigengene and merged similar modules (abline = 0.25). A module is a group of genes with high topological overlap similarity, meaning that genes in the same module are highly co-expressed. Finally, we calculated the correlation between different modules and the clinical data to identify modules with significant clinical relevance.

### 3.4. GO and KEGG Analysis

To understand the biological function or pathway involved with key genes of DFU, we utilized packages such as ggplot2 and clusterProfiler in the R project to conduct GO and KEGG enrichment analysis of 388 key genes and visualized the results. GO analysis includes cell component (CC), biological process (BP), and molecular function (MF).

### 3.5. Protein-Protein Interaction Network and Identification of Hub Genes

The STRING website was used to search the protein-protein interaction (PPI) relationships of key genes. Cytoscape software was used to establish and visualize PPI networks based on the PPI relationships. The cytoHubba plugin in Cytoscape software was used to calculate the number of adjacent nodes of these key genes and score them (based on the method of Degree). We sorted the genes from high to low and identified the top 15 genes with the highest scores as hub genes.

### 3.6. Support Vector Machine-Recursive Feature Elimination

Support vector machine-recursive feature elimination (SVM-RFE), a supervised machine-learning algorithm, was used to identify the diagnostic biomarkers with superior discriminative ability in DFU. The e1071, kernlab, and caret packages in R software were used for SVM-RFE analysis.

### 3.7. Immunocyte Infiltration Analysis

The e1071 and preprocessCore packages in the R project were used to calculate the relative content of each immune cell in each sample based on the GSE80178 dataset. We utilized the pheatmap package to draw the heat map, and the reshape, ggpubr, and ggExtra packages were used to explore the correlation between the expression of hub genes and immune cell levels.

### 3.8. Single-Cell Transcriptomic and Intercellular Communication Analysis

We detected differences in cellular landscape and transcriptome between DFU healers (n = 9) and DFU non-healers (n = 5) based on the single-cell DFU dataset (GSE165816). The SingleR package was used to conduct single-cell transcriptomic analysis. We first normalized the data and integrated Seurat objects into a merged dataset. Harmony was used to correct batch effects in scRNA-seq data integration. The principal component analysis algorithm was used to reduce data dimensions. We clustered cells using FindNeighbors and FindClusters and identified the DEG of cell populations by using FindAllMarkers. Hematopoietic.RData, ImmuneCellExpressionData.Rdata, ImmGenData.Rdata, and Human_All.RData were used to automatically annotate cells. We conducted the intercellular communication analysis using the Sqjin/CellChat package. CellChatDB.human was used to establish a network for ligand-receptor crosstalk. We filtered out intercellular communication expressed by fewer than 10 cells. NetAnalysis_Continuation was used to calculate the contribution of each ligand-receptor to the entire signaling pathway. PlotGeneExpression was used to visualize the expression of ligands and receptors in signaling pathways. NetAnalysis_TCentrality and netAnalysis_SignalingRole_Network were used to identify senders, receivers, intermediaries, and influencers in certain networks. The role of hub genes in different cell populations and intercellular communication was further analyzed.

### 3.9. Multiplex Immunohistochemical Analysis

We collected foot skin tissues from 3 DFU healers and ulcer tissues from 3 DFU non-healers at the Plastic Surgery Hospital of the Chinese Academy of Medical Sciences and Peking Union Medical College. We first dehydrated the tissue samples, embedded them in paraffin, and sliced them. We then dewaxed the slices and used ethanol with gradient solubility for hydration. Subsequently, antigen repair was carried out by boiling the slices in the repair solution at 95°C for 15 - 20 minutes. We used a 10% BSA blocking solution for blocking and incubated the slices with the primary antibody at 4°C overnight. Antibodies against CD90 (Abcam, 1:400, Cambridgeshire, UK), CD44 (Abcam, 1:1000, Cambridgeshire, UK), E-cadherin (Abcam, 1:100, Cambridgeshire, UK), FGF7 [cell signaling technology (CST), 1:400, USA], and FGFR1 (Affinity Biosciences, 1:500, USA) were used. After incubating with the secondary antibody at room temperature for 50 minutes, we added TSA fluorescent dye reaction solution onto the slices and repeated the steps after antigen repair (if double or triple labeled, change to other dyes and continue labeling). After using DAPI to stain the cell nucleus, we observed and took photos of slices under the OLYMPUS microscope. This study was approved by the Medical Ethics Committee of the Plastic Surgery Hospital of the Chinese Academy of Medical Sciences and Peking Union Medical College (2024-218), and informed consent was obtained from all patients.

### 3.10. Software and Package Versions

The analysis was performed using R version 4.3.0. DEGs were identified using the limma package (version 3.58.1) in R, with the threshold set at fold change > 2 and adjusted P-value < 0.05. For WGCNA analysis, the version of the WGCNA package is 1.73, and the soft threshold is 18 (R^2^ = 0.721, truncated R^2^ = 0.842, slope = -0.983). For the PPI network, STRING and Cytoscape software version 3.10.0 were used. For SVM-RFE, the e1071 (version 1.7.16), kernlab (version 0.9.33), and caret (version 7.0.1) packages were used. For single-cell analysis, the singleR (version 2.4.1) and CellChat (version 1.6.1) packages were used. For immunocyte infiltration analysis, the e1071 (version 1.7.16) and preprocessCore (version 1.64.0) packages were used.

## 4. Results

### 4.1. Identification of Key Genes in Diabetic Foot Ulcers

**Figure 1.**
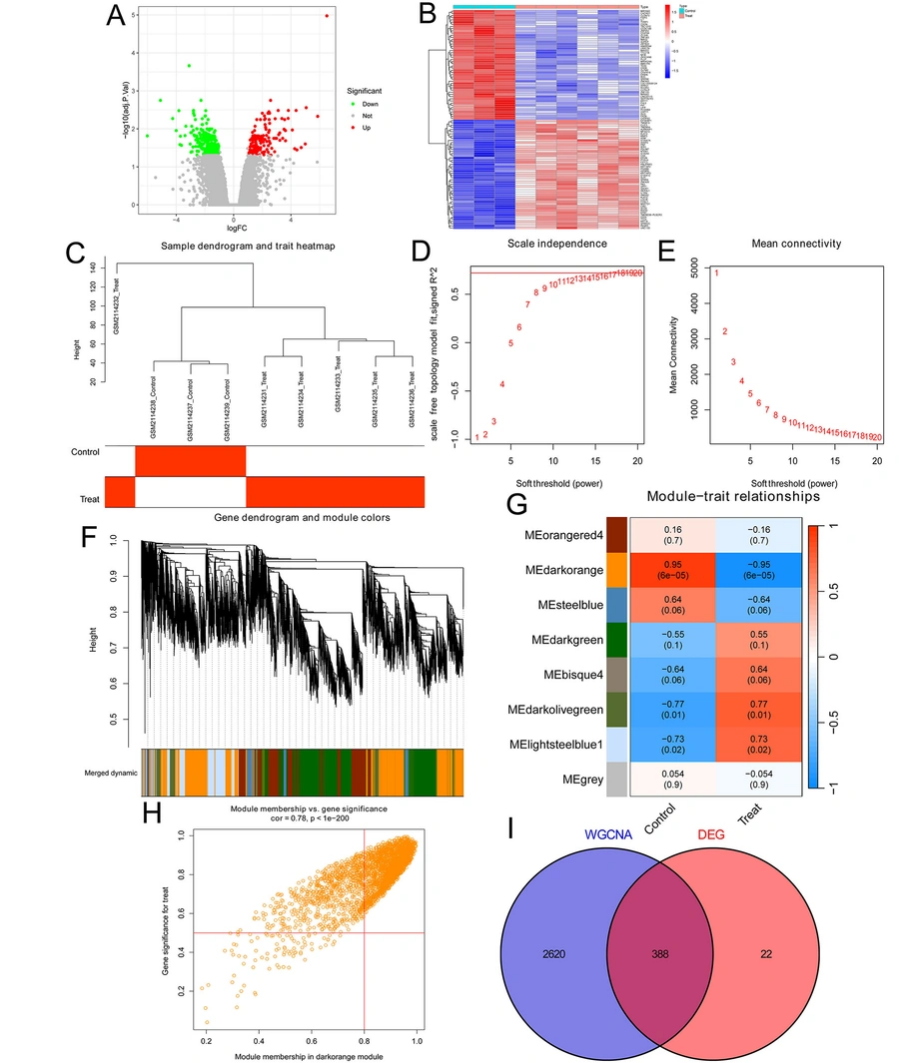
Identification of key genes in diabetic foot ulcers (DFUs): A, the volcano plot showed all DEGs with over a 2.0-fold change between 3 diabetic foot skin (DFS) tissues and 6 DFU tissues in the GSE80178 DFU datasets. 142 upregulated genes are marked in red, and 268 downregulated genes are marked in green; B, the cluster heat map showed the expression of the top 50 and bottom 50 DEGs; C, the sample clustering tree of 3 DFS tissues and 6 DFU tissues; D, analysis of the Scale-Free Index for various soft-threshold powers; E, analysis of the mean connectivity for various soft-threshold powers; F, dendrogram of genes clustered based on the measurement of dissimilarity. The color band shows the results of identifying modules and merging similar modules; G, heatmap of the correlation between the module eigengenes and DFUs; H, analysis of gene significance for DFUs and module membership in the dark orange module; I, Venn diagram showing the intersection of the DEGs in the DFUs and the DFU-associated genes in the dark orange module.

We first analyzed the genomic profiling in three DFS tissues and six DFU tissues using previously published DFU datasets (GEO#GSE80178). We found 410 genes (including 142 up-regulated genes and 268 down-regulated genes) with over a 2.0-fold change between DFU and DFS (threshold: Fold change > 2 and adjusted P < 0.05; Appendix 4 in Supplementary File). The volcano plot showed all DEGs in DFU (Figure 1A), and the top and bottom 50 DEGs were shown in the cluster heat map (Figure 1B). We next conducted the WGCNA analysis through the WGCNA package in the R project based on the GSE80178 datasets to identify DFU-associated genes. After normalization and correction of data, a total of 9376 genes were included in the analysis. Subsequently, we clustered the samples and drew a sample clustering tree (Figure 1C). We set the soft threshold to 18 (the value reaching a plateau, R^2^ = 0.721, truncated R^2^ = 0.842, slope = -0.983) to construct a scale-free network (Figures 1D and E), and the WGCNA was conducted according to the steps described in the WGCNA section of the methods. A total of eight modules and the genes they contained were identified (Figure 1F). We discovered that the dark orange module was most associated with DFU (R = 0.78 and P < 0.05; Figures 1G and H), thus genes in this module (Appendix 5 in Supplementary File) were identified as the DFU-associated genes. Finally, we took the intersection of the DEGs in the DFU and the DFU-associated genes in the dark orange module, resulting in 388 key genes (Figure 1I). The list of key genes is shown in Appendix 6 in Supplementary File, and these genes were used for further analysis.

### 4.2. Constructing Protein-Protein Interaction Network and Identifying Specific Biomarkers in Diabetic Foot Ulcers

**Figure 2.**
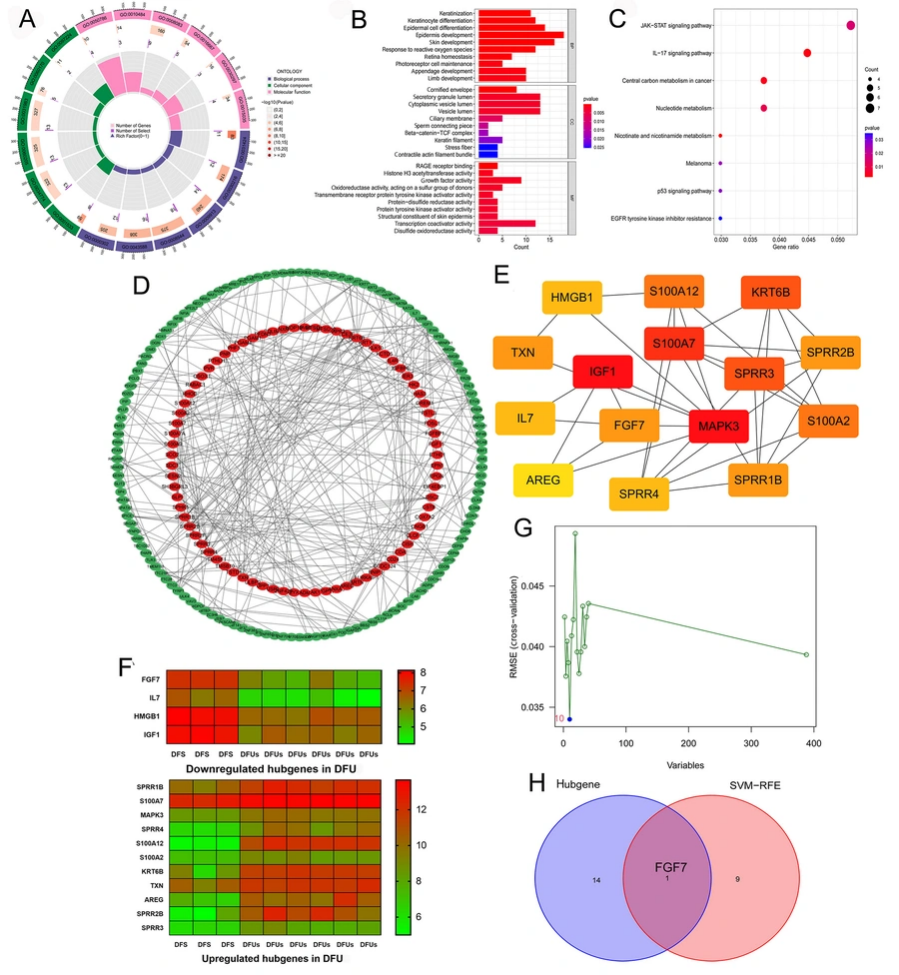
Constructing protein-protein interaction (PPI) network and identifying specific biomarkers in diabetic foot ulcers (DFUs): A, GO enrichment analysis of the 388 key genes. BP, CC, and molecular function (MF) are displayed in different colors, respectively. All genes and the number of key genes of DFUs involved in different cell functions are also displayed; B, GO enrichment analysis of the 388 key genes, showing the top ten cell functions of BP, CC, and MF involved in these key genes; C, KEGG enrichment analysis of the 388 key genes showcased the signaling pathways involved in these key genes; D, the PPI network of the 388 key genes was constructed using the STRING website and Cytoscape software; E, the cytoHubba plugin in Cytoscape software identified the 15 network hub genes, and the network diagram of these hub genes is shown; F, the expression of 15 hub genes in DFS and DFUs from the GSE80178 datasets; G, support vector machine-recursive feature elimination (SVM-RFE) was used to screen out the potential diagnostic biomarkers of DFUs among 388 key genes, and 10 genes were identified as diagnostic biomarkers for DFUs; H, the intersection of the results of hub genes and SVM-RFE.

We used the related package in the R project to conduct an enrichment analysis of GO (including CC, BP, and MF) and KEGG on the 388 key genes. As shown in Figure 2A and B, the BP group genes were mainly enriched in epidermis development, epidermal cell differentiation, skin development, keratinocyte differentiation, keratinization, and so on. In the CC group, genes were mainly associated with secretory granule lumen, cytoplasmic vesicle lumen, vesicle lumen, and cornified envelope. Meanwhile, genes in the MF group were mainly related to transcription coactivator activity, growth factor activity, RAGE receptor binding, structural constituent of skin epidermis, and transmembrane receptor protein tyrosine kinase activator activity. In addition, KEGG analysis showed that these key genes were significantly related to the JAK-STAT signaling pathway, IL-17 signaling pathway, and central carbon metabolism in cancer (Figure 2C). These results suggest that these key genes may be involved in the healing process of DFU and intercellular communication.

The STRING website and Cytoscape software were used to build the PPI network based on the 388 key genes, and the results are presented in Figure 2D and Appendix 7 in Supplementary File. Next, we scored each gene using the cytoHubba plugin in Cytoscape software, and the overall results of the scoring are displayed in Appendix 8 in Supplementary File. We selected the 15 genes with the highest scores as the network hub genes based on the scores (the number of neighboring nodes), including HMGB1, S100A12, KRT6B, TXN, IGF1, S100A7, SPRR2B, IL7, FGF7, MAPK3, S100A2, AREG, SPRR4, SPRR1B (Appendix 9 in Supplementary File). The network diagram of hub genes is shown in Figure 2E. The expression of 15 hub genes in DFS and DFU from GSE80178 datasets is shown in Figure 2F. 

Moreover, we used SVM-RFE to screen out the potential diagnostic biomarkers of DFU among those 388 key genes, and 10 genes were identified as diagnostic biomarkers for DFU (FGF7, GTF2IRD2, KLK10, MIR573, NEO1, BCL11A, PLN, ZNF814, SLIT3, CFAP418) (Figure 2 and Appendix 10 in Supplementary File). When taking the intersection of the results of hub genes and SVM-RFE, we obtained only a unique gene, which is FGF7 (Figure 2H), which may be the specific biomarker in DFU. We found that FGF7 is lowly expressed in DFU (Figure 2F). 

### 4.3. Single-Cell Transcriptomic and Immune Cell Analysis of Diabetic Foot Ulcers

**Figure 3.**
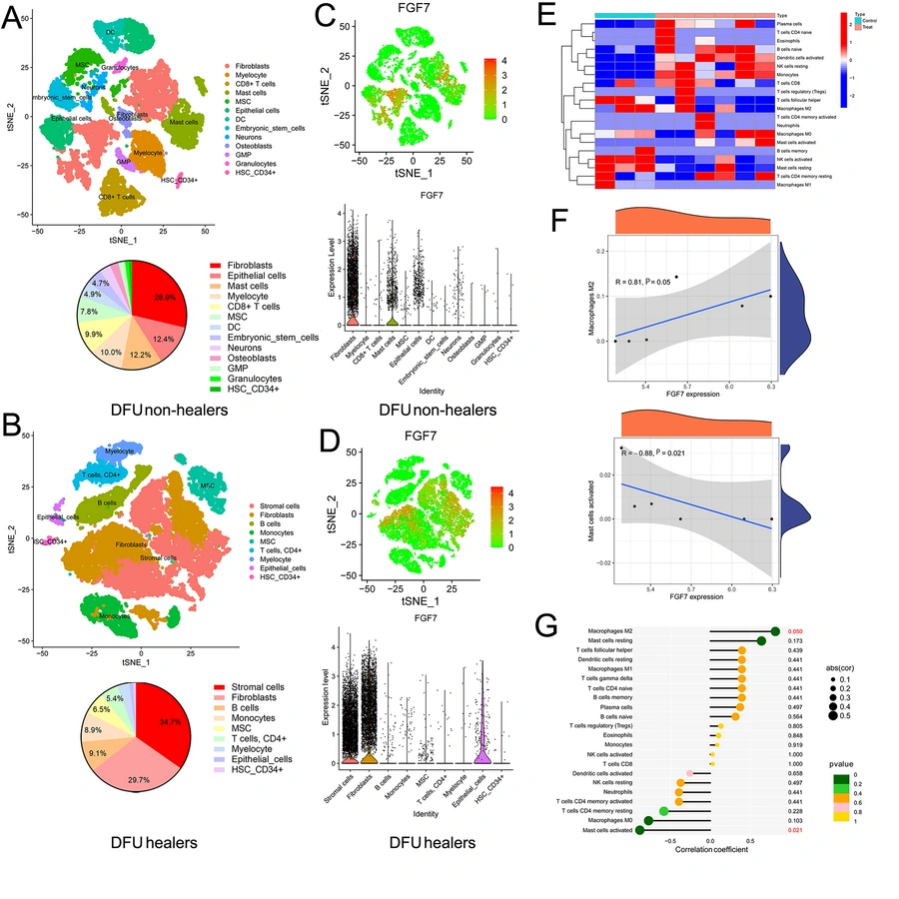
Single-cell transcriptomic and immune cell analysis of diabetic foot ulcers (DFUs): A, the cellular landscape of DFU non-healers by analyzing single-cell transcriptomic datasets of DFUs (GSE165816). The cell clusters were annotated according to various canonical markers based on the expression of specific markers, and we also calculated the proportion of different cells; B, the cellular landscape of DFU healers. We annotated various cell clusters according to the expression of specific markers and calculated the proportion of different cells; C, the expression of FGF7 in different cells of DFU non-healers; D, the expression of FGF7 in different cells of DFU healers; E, analysis of immune cell levels in 3 diabetic foot skin (DFS) tissues and 6 DFU tissues based on DFU datasets; F, correlation analysis between the expression of FGF7 and the level of M2 macrophages, and the correlation between the expression of FGF7 and the level of activated mast cells; G, the lollipop chart shows the correlation between the expression of FGF7 and all immune cells.

Single-cell transcriptomic analysis reveals the transcriptomic landscape of individual cells in tissues, providing greater insight into cellular function and disease progression. Therefore, we analyzed the single-cell transcriptomic datasets of DFU (GEO#GSE165816) through the SingleR package in the R project to explore the differences in transcriptome and cellular landscape between DFU healers (n = 9) and DFU non-healers (n = 5). The t-SNE plots with clustering metrics in DFU healers and DFU non-healers are shown in Appendix 1 in Supplementary File. As shown in Figure 3A and B, the DFU healers had a higher proportion of unclassified stromal cells (34.7%) (many different types of stromal cells can promote wound healing) compared to DFU non-healers. Additionally, in terms of immune cells, the DFU healers showed a significantly higher proportion of B-cells (9.1%) and monocytes (8.9%), while DFU non-healers showed more mast cells (12.2%), CD8+ T-cells (9.9%), and myelocytes (10.0%). We then analyzed the expression of hub genes in different cells of DFU (Appendix 2A and B in Supplementary File). Particularly, FGF7 is mainly expressed in fibroblasts and has limited expression in mast cells and epithelial cells in DFU non-healers, while it is mainly expressed in stromal cells and fibroblasts in DFU healers (Figures 3C and D).

To further explore the role of FGF7 in the immune cells of DFU, we detected the levels of immune cells in DFS and DFU based on the GSE80178 database. Many immune cells are at higher levels in DFU (Figure 3E). Moreover, the expression level of FGF7 was positively correlated with Macrophages M2 (activated macrophages with anti-inflammatory properties, which could promote remission of the inflammatory phase and the wound to enter the proliferation phase), but negatively correlated with activated mast cells (which mainly act on the inflammatory stage of the wound, and their continuous effect is related to the chronic wound) (Figures 3F and G). These results indicated that FGF7 can not only affect the wound healing of DFU through stromal cells and fibroblasts but also participate in the immune response of the wound to promote healing in DFU.

### 4.4. Deciphering Intercellular Communication in Diabetic Foot Ulcers Healers and Diabetic Foot Ulcers Non-healers

**Figure 4.**
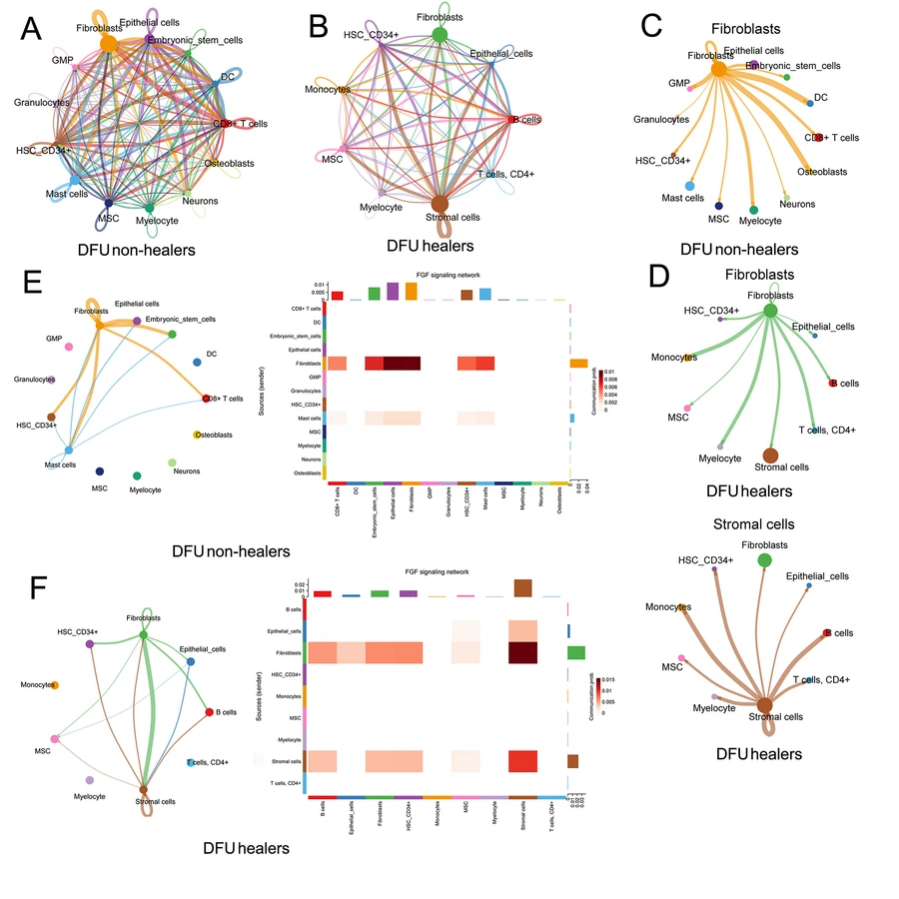
Deciphering intercellular communication in diabetic foot ulcer (DFU) healers and DFU non-healers: A, all the potential intercellular communication networks in DFU non-healers; B, all intercellular communication networks in DFU healers; C, the intercellular communication pathways between fibroblasts and other cells in DFU non-healers; D, the intercellular communication pathways between fibroblasts and stromal cells and other cells in DFU healers; E, intercellular fibroblast growth factor (FGF) signaling network involving FGF7 in DFU non-healers; F, intercellular FGF signaling network involving FGF7 in DFU healers.

Further, we analyzed the co-expression of ligand, receptor, and target genes in DFU to detect possible intercellular communication using the sqjin/CellChat package in the R project. We first established all the potential intercellular communication networks in DFU healers and DFU non-healers (Figures 4A and B) and showed all intercellular pathways and corresponding ligand-receptor pairs (Appendix 3A and B in Supplementary File). It is expected that fibroblasts are the most numerous cells involved in the cellular communication of DFU non-healers, and stromal cells and fibroblasts also account for the highest proportion in DFU healers’ cellular communication (Figures 4A and B). The communication pathways between fibroblasts and stromal cells and other cells are shown in Figures 4C and D. We then detected whether hub genes are involved in cellular communication and found that FGF7 primarily participated in the intercellular FGF pathway involved in fibroblasts and stromal cells, and showed the intercellular FGF pathway in DFU (Figures 4E and F).

### 4.5. Exploring the Fibroblast Growth Factor Pathway of Diabetic Foot Ulcers Involved by FGF7 and mIHC Validation

**Figure 5.**
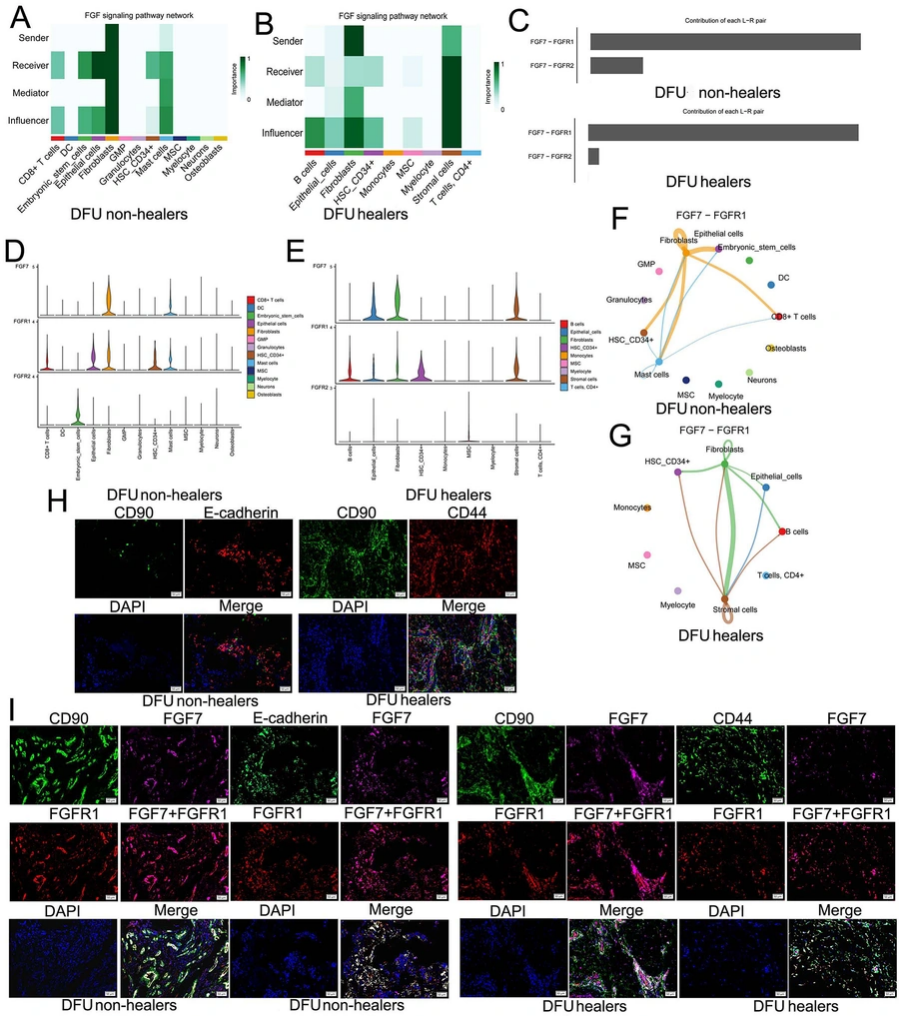
Exploring the fibroblast growth factor (FGF) pathway of diabetic foot ulcers (DFUs) involved by FGF7 and mIHC validation: A, in DFU non-healers, we analyzed the roles of different cells in the FGF signaling pathway network, including sender, receiver, influencer, and mediator; B, we analyzed the roles of different cells in the FGF signaling pathway network of DFU healers, including sender, receiver, influencer, and mediator; C, the contribution of FGF7-FGFR1 and FGF7-FGFR2 in DFUs; D, expression of FGF7 and its receptor FGFR1 and FGFR2 in DFU non-healers; E, expression of FGF7 and its receptor FGFR1 and FGFR2 in DFU healers; F, intercellular communication involved in the FGF7-FGFR1 signaling pathway in DFU non-healers; G, intercellular communication involved in the FGF7-FGFR1 signaling pathway in DFU healers; H, mIHC assay confirmed the presence of crosstalk in fibroblasts, epithelial cells, and stromal cells; I, in DFU non-healers and DFU healers, the mIHC assay showed that FGF7 and FGFR1 co-expressed in CD90+ fibroblasts, E-cadherin+ epithelial cells, and CD44+ stromal cells.

We further analyzed the intercellular FGF pathways that FGF7 participates in. In DFU non-healers, fibroblasts primarily act as senders to send ligands, with fibroblasts themselves and epithelial cells as the main receivers of signal molecules compared to other cells (Figure 5A). In DFU healers, fibroblasts are the main senders, stromal cells also have a limited role in sending signals, and stromal cells are the primary signal receivers compared to other cells (Figure 5B). Moreover, FGF7-FGFR1 is the ligand-receptor pair that plays a major role in both DFU healers and DFU non-healers (Figure 5C). Meanwhile, in DFU non-healers, FGF7 is mainly expressed in fibroblasts, and its receptor FGFR1 is expressed in fibroblasts, epithelial cells, CD8+ T-cells, HSC_CD34+, and mast cells (Figure 5D). In DFU healers, FGF7 is expressed in fibroblasts, stromal cells, and epithelial cells, and the receptor FGFR1 is expressed in fibroblasts, stromal cells, epithelial cells, B-cells, and HSC_CD34+ (Figure 5E). In addition, it should be pointed out that the FGF7-FGFR1 pathway mainly involves the autocrine pathway of fibroblasts and its interaction with epithelial cells in DFU non-healers (Figure 5F). For DFU healers, FGF7-FGFR1 is mainly involved in the communication between fibroblasts and stromal cells, as well as the self-communication of stromal cells (Figure 5G). 

Furthermore, the mIHC assay was used to confirm the results of bioinformatics analysis in tissue samples. The aggregation of fibroblasts (CD90 is a cellular marker) and epithelial cells (E-cadherin is a cellular marker) was found in DFU non-healers, while fibroblasts (CD90 is a cellular marker) and stromal cells (CD44 is a cellular marker) were also proven to aggregate in DFU healers (Figure 5H). These results indicated the presence of crosstalk between these cells. In DFU non-healers, both CD90+ fibroblasts and E-cadherin+ epithelial cells co-express FGF7 and FGFR1; in DFU healers, FGF7 and FGFR1 are also co-expressed in CD90+ fibroblasts and CD44+ stromal cells (Figure 5I). 

## 5. Discussion

As one of the most serious complications of diabetes, DFU have a long hospital stay and a high recurrence rate, causing great pain and a serious financial burden to individuals (31, 32). Therefore, it is necessary to deeply understand the pathogenesis of DFU. In this study, we first analyzed the GSE80178 DFU datasets to conduct gene differential expression analysis and WGCNA analysis, and a total of 388 key genes have been identified. GO and KEGG analysis showed that these genes were mainly enriched in some pathways that may be related to DFU healing and intercellular communication. Next, we built the PPI network of 388 key genes through the STRING website and Cytoscape software and identified 15 hub genes using the cytoHubba plugin. Moreover, we screened out 10 genes as potential diagnostic biomarkers for DFU through SVM-RFE and intersected them with the 15 hub genes to obtain specific biomarkers (FGF7) in DFU.

The formation of chronic wounds in DFU is due to the damage to the wound healing process of diabetes patients caused by many factors (11). Many different types of cells play important roles in different stages of wound healing (12, 15). Stromal cells play a significant role in the process of wound healing. For instance, mesenchymal stromal cells can induce angiogenesis, re-epithelialization, and formation of granulation tissue to promote wound closure (33). Adipose tissue-derived stromal cells can secrete various cytokines and growth factors and differentiate into skin cells (34). To gain a deeper understanding of the functions of different cells in DFU and their impact on DFU progression, we analyzed the single-cell transcriptomic datasets of DFU. We found that DFU healers had a higher proportion of stromal cells compared to non-healers. FGF7 is mainly expressed in fibroblasts in DFU non-healers and enriched in stromal cells and fibroblasts in DFU healers. Additionally, there is a significant difference in immune cells between DFU healers and DFU non-healers. To further explore the role of immune cells in the inflammatory responses of DFU, we analyzed the GSE80178 database and found many immune cells are at higher levels in DFU. It has been confirmed that DFU exhibit a chronic pro-inflammatory state, such as macrophages continuing to maintain a pro-inflammatory state, the production of pro-inflammatory cytokines, and an inflammatory response enhanced by neutrophils (35-37). Moreover, the level of FGF7 was positively correlated with M2 anti-inflammatory macrophages but negatively correlated with activated pro-inflammatory mast cells. These results indicated that FGF7 may alleviate the chronic pro-inflammatory state of DFU and facilitate wound healing by promoting the wound to enter the proliferative stage through activating M2 macrophages. Notably, our pathway analysis revealed that FGF7 plays a critical role in DFU healing, which is compatible with previous work demonstrating that FGF7 promotes tissue repair in chronic wounds (38).

We analyzed DFU intercellular communication using single-cell transcriptomics. In non-healers, fibroblasts mainly send ligands, with fibroblasts and epithelial cells as primary receivers. In healers, fibroblasts are key senders, stromal cells secrete ligands, and stromal cells are the main receivers. FGF7 is primarily involved in FGF signaling between fibroblasts and stromal cells. Cellular communication and mIHC analysis showed that FGF7-FGFR1 in non-healers involves fibroblast autocrine signaling and communication with epithelial cells, while in healers, it primarily mediates fibroblast-stromal cell communication and stromal cell autocrine signaling.

Previous studies on the sequencing analysis of chronic wounds have played a significant role in elucidating the mechanisms of wound onset and healing. Scholars have conducted single-cell sequencing on DFU, thereby offering valuable resources for the study of diabetic foot and wound healing (13). Based on this database, we combined multi-dimensional transcriptomic analysis and histological validation to further reveal the role of FGF7 in DFU. Others have used a combination of metabolomics and transcriptomics to reveal the mechanisms of non-diabetic chronic wounds (39). Compared to these studies, we combined single-cell sequencing, machine learning algorithms, immunological cell analysis, and histological validation, thereby providing a multidimensional confirmation of the important role of the FGF7 signaling pathway in the healing of DFU. Currently, scholars have explored the application of the FGF family in therapy to promote the healing of chronic wounds (30). Additionally, some researchers have immobilized FGF on poly(xylitol dodecanedioic acid) polymer for tissue regeneration (40). These studies demonstrate the significant clinical potential of FGF in accelerating wound healing. Future research based on our findings could develop localized drugs targeting FGF7 to enhance DFU healing.

However, this study still has some limitations. Firstly, the analysis was based on public datasets, which may lack comprehensive clinical annotations (such as infected, ischemic, neuropathic). Secondly, the sample size was relatively small. In the future, we will deepen the clinical application research of FGF7, such as detecting the levels of FGF7 in the serum or wound fluid of DFU patients, and exploring the relationship between FGF7 expression and the severity or healing time of DFU.

In summary, we have proved that the FGF7-FGFR1 pathway plays an important role in the intercellular communication of fibroblasts and stromal cells during DFU wound healing. In the future, topically acting drugs targeting FGF7 could be developed based on our findings to treat DFU and accelerate wound healing.

## Data Availability

The dataset presented in the study is available on request from the corresponding author during submission or after publication.
